# Protein profile and protein interaction network of *Moniliophthora perniciosa* basidiospores

**DOI:** 10.1186/s12866-016-0753-0

**Published:** 2016-06-24

**Authors:** Joise Hander Mares, Karina Peres Gramacho, Everton Cruz dos Santos, André da Silva Santiago, Edson Mário de Andrade Silva, Fátima Cerqueira Alvim, Carlos Priminho Pirovani

**Affiliations:** Departamento de Ciências Biológicas, Universidade Estadual de Santa Cruz, Ilhéus, Bahia Brazil; Centro de Biologia Molecular e Engenharia Genética (CBMEG), Universidade Estadual de Campinas, Campinas, SP Brazil; Centro de Pesquisas do Cacau - CEPEC, Comissão Executiva do Plano da Lavoura Cacaueira- CEPLAC, Ilhéus, Bahia Brazil

**Keywords:** Basidiospores, Interaction network, Mass spectrometry, Proteomics, Witches’ broom

## Abstract

**Background:**

Witches’ broom, a disease caused by the basidiomycete *Moniliophthora perniciosa*, is considered to be the most important disease of the cocoa crop in Bahia, an area in the Brazilian Amazon, and also in the other countries where it is found. *M. perniciosa* germ tubes may penetrate into the host through intact or natural openings in the cuticle surface, in epidermis cell junctions, at the base of trichomes, or through the stomata. Despite its relevance to the fungal life cycle, basidiospore biology has not been extensively investigated. In this study, our goal was to optimize techniques for producing basidiospores for protein extraction, and to produce the first proteomics analysis map of ungerminated basidiospores. We then presented a protein interaction network by using *Ustilago maydis* as a model.

**Results:**

The average pileus area ranged from 17.35 to 211.24 mm^2^. The minimum and maximum productivity were 23,200 and 6,666,667 basidiospores per basidiome, respectively. The protein yield in micrograms per million basidiospores were approximately 0.161; 2.307, and 3.582 for germination times of 0, 2, and 4 h after germination, respectively. A total of 178 proteins were identified through mass spectrometry. These proteins were classified according to their molecular function and their involvement in biological processes such as cellular energy production, oxidative metabolism, stress, protein synthesis, and protein folding. Furthermore, to better understand the expression pattern, signaling, and interaction events of spore proteins, we presented an interaction network using orthologous proteins from *Ustilago maydis* as a model. Most of the orthologous proteins that were identified in this study were not clustered in the network, but several of them play a very important role in hypha development and branching.

**Conclusions:**

The quantities of basidiospores 7 × 10^9^; 5.2 × 10^8^, and 6.7 × 10^8^ were sufficient to obtain enough protein mass for the three 2D-PAGE replicates, for the 0, 2, and 4 h-treatments, respectively. The protein extraction method that is based on sedimentation, followed by sonication with SDS-dense buffer, and phenolic extraction, which was utilized in this study, was effective, presenting a satisfactory resolution and reproducibility for *M. perniciosa* basidiospores. This report constitutes the first comprehensive study of protein expression during the ungerminated stage of the *M. perniciosa* basidiospore. Identification of the spots observed in the reference gel enabled us to know the main molecular interactions involved in the initial metabolic processes of fungal development.

**Electronic supplementary material:**

The online version of this article (doi:10.1186/s12866-016-0753-0) contains supplementary material, which is available to authorized users.

## Background

Cocoa beans (*Theobroma cacao* L.) are the raw material for chocolate manufacturing. In South and Central America, production of these beans is affected by several diseases, mainly caused by fungi [1], such as witches’ broom disease caused by the basidiomycota *Moniliophthora perniciosa* [2]. Witches’ broom is considered to be the most important disease in tropical America including Bahia, the most important cocoa-producing region in Brazil [3]. The life cycle starts with the release of basidiospores, which are the only infective propagules of the fungus. Basidiospores emerge from basidiomes produced on dry brooms, which are necrotized branches [4]. Basidiospores (n), meiospores of *M. perniciosa*, are easily carried over short distances by wind and can lead to an epidemic. Early processes of infection include basidiospore attachment, germination, and the formation of a germ tube. The germ tubes differentiate into club-shaped hyphae, which can penetrate into natural openings or wounds. Natural openings include the cuticle surface, the epidermis cell junctions, the base of trichomes, and the stomata [5]. Within 24 h after spore germination, biotrophic hyphae can be seen in the apoplasts of infected tissues, inducing macroscopic modifications such as the formation of green brooms.

Since basidiospores are the critical inoculum for infection that cocoa encounters in the field, studies that directly examine these spores are critical. In vitro basidiospore production requires basidiospore inoculum free of other contaminating organisms. Protocols for in vitro and in vivo production of *M. perniciosa* basidiospores have been established [6, 7]. The current techniques to cast basidiospores [8] permit storage of both precise and clean basidiospores as required for efficient studies, i.e. inhibition tests of basidiospore germination, identifying volatile compounds produced by endophyte fungi of cacao [9], screening of cacao genotypes for witches’ broom disease [10], and studying fungal biology [11], among others.

The genome and transcriptome resources for *M. perniciosa* have been generated [12, 13]. These data have been used in several studies to reveal many aspects of the *Theobroma cacao* vs. *M. perniciosa* interaction. Furthermore, a representative cDNA library of the host-pathogen interaction is also available for the identification of pathogen and host genes involved in pathogenicity and resistance mechanisms [14]. Several studies support efforts to characterize proteins related to metabolic routes during fungal infection [15–21]. For example, the effects of TcPR10, a protein with ribonuclease activity and potential to induce proteome variations in the mycelium, were examined in *M. perniciosa* [22]. Recently, studies identified genes differentially expressed in *M. perniciosa* basidiospores after exposure to *Theobroma cacao* from high-resolution transcriptomic data [23].

Proteomic studies are needed to broaden the knowledge of *M. perniciosa* metabolism during the initial life cycle phase to enhance the control strategies for this plant pathogen. For instance, proteomic analysis may help us to understand the expression, function, and regulation of specific groups of proteins encoded by the fungal genome [24]. Using this approach, several proteins involved in spore metabolism of plant pathogenic fungi *Blumeria graminis* f.sp. *hordei* [25], *Aspergillus nidulans* [26], and *Colletotrichum acutatum* have been identified [27]. However, there are still very few molecular studies using *M. perniciosa* [23], probably due to the difficulty to cast and store the high volume of basidiospores needed for protein extraction and analysis.

Data generated by molecular analyses such as large-scale genomics, transcriptomics, and proteomics may be used to build biological system models through computerized analyses offered by systems biology [28]. Systems biology may translate the complex molecular interactions between genotype and phenotype that exist in biological systems. The idea behind systems biology is that cell networks and biological systems are the bridges between genotypes and phenotypes [29]. Inside those networks, the centrality analysis ranks elements in order to identify the most important components [30]. Central vertices in a network are those nodes, which have a functional or structural importance, and they may influence many other vertices through short and direct pathways [31]. Therefore, centrality measures, in general, capture the structural importance of a vertex regarding the rest of the network. According to the importance levels in an interaction, proteins may be defined as nonhub-non-bottleneck, hub-nonbottleneck, nonhub-bottleneck, and hub-bottleneck [32]. According to Yu et al., in both interaction and regulation networks, bottleneck and hub-bottleneck proteins tend to be essential proteins with a high degree of significance [32].

Interaction analyses of microbial proteins have been performed in fungi, mainly yeast. Yeast interaction networks are now evident and they may be explored, serving as an exemplar model for the most complex eukaryotes [33, 34]. A polygalacturonase interaction network, MpPG1 and MpPG2, of *M. perniciosa* has been established using orthologous sequences of *Neurospora crassa* as a model [35]. However, studies related to plant pathogen fungal spores are scarce.

In this study, we monitored spore production and estimated the number of basidiomes and basidiospores, which are produced in the brooms of a susceptible, diseased cacao genotype named Catongo, allowing us to obtain sufficient protein yields for proteomic studies of germination onset. The protein profile of ungerminated spores was established via SDS-PAGE. A total of 178 proteins were identified through mass spectrometry MS/MS. Furthermore, in order to better understand the expression pattern, signaling, and interaction events of spore proteins, we presented an interaction network using *Ustilago maydis* as a model.

## Methods

For organization purposes, the workflow was prepared using a flowchart of steps (Additional file 1: Figure S1).

### Basidiospore production

Approximately 300 dry brooms (diseased cacao branches) of a susceptible genotype were randomly collected in a field of the Arnaldo Medeiros Experimental Station at the Cocoa Research Center, CEPLAC/CEPEC-Ilhéus, BA, Brazil. Brooms were superficially disinfected with 0.5 % sodium hypochlorite, hung individually within a cabinet, and subjected to a daily regime of 8 h wet/16 h dry, at a temperature of 25 ° C ± 3 °C, with 100 % humidity from August 2010 to July 2011. Basidiospore concentrations were established prior to storage with aid of a Neubauer chamber and by observation under an optical microscope (Bioval L2000A, 40× magnification). Basidiospores were stored in liquid N_2_, according to methods described by Frias [4, 8].

Before basidiospore production, basidiome size was analyzed. Pilei from 2081 basidiomes were measured with the aid of a universal caliper and were divided in 24 groups according to pileus area. The number of pilei per sample ranged from 15 to 204 (Additional file 2: Table S1). The average productivity per pileus area and average productivity per basidiome were established for each sample (Additional file 2: Table S1).

### Basidiospore germination and protein extraction

For protein extraction and germination viability, basidiospores were defrosted on ice, diluted with distilled water to reduce the glycerol concentration to 3 % [8], and were incubated in the dark at 25 °C. The samples were placed between two glass slides, and germination rates and length of germ tubes were monitored and measured under an optical microscope (Bioval L2000A, 40×).

**Fig. 1:**
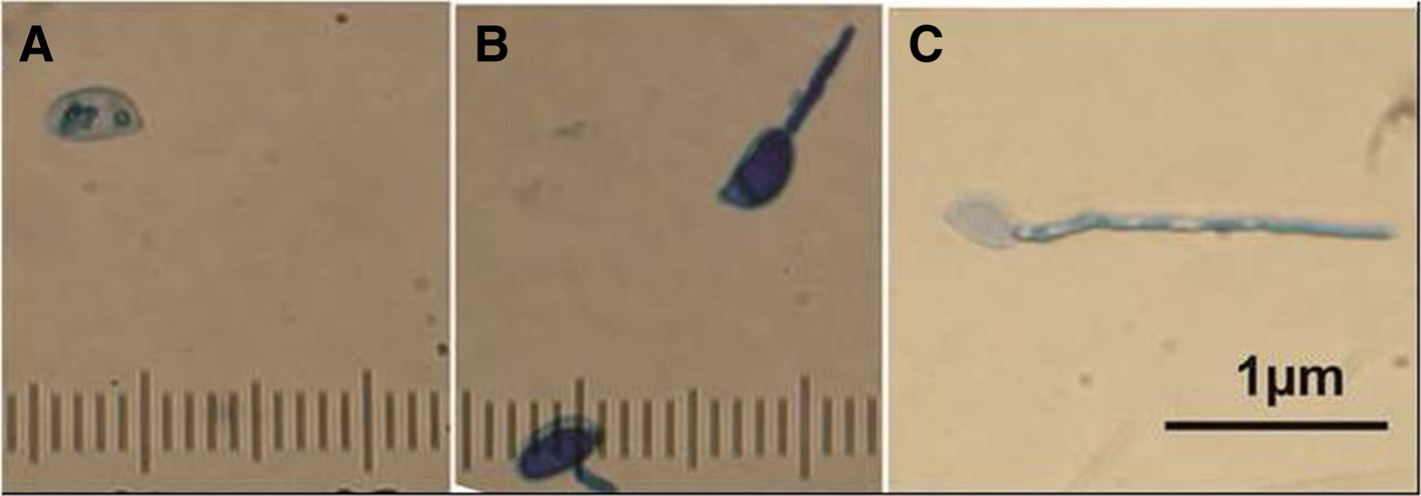
Morphological differences observed during *M. perniciosa* basidiospore germination: **a** ungerminated basidiospore (0 h); **b**) 2 and **c**) 4 h after basidiospore germination. Basidiospores were visualized after Cotton Blue staining

For protein extraction, basidiospores were allowed to germinate; then, they were centrifuged and casted in 3 stages representative of the initial germination phase after germination: (i) ungerminated (0 h treatment), (ii) 2 h after germination (HAG), and (iii) 4 HAG (Fig. 1). The basidiospore germ tube growth was measured using the formula below (also see Fig. 3).

$$ \mathrm{Germ}\ \mathrm{tube}\ \mathrm{growth}=\left(\mathrm{spore}\ \mathrm{diameter}+\mathrm{germ}\ \mathrm{tube}\ \mathrm{length}\right)/\mathrm{spore}\ \mathrm{diameter} $$

### Protein extraction

After the appropriate incubation period, basidiospores were sedimented by centrifugation at 5,000 × *g* for 20 min. The supernatant was discarded and the precipitate was washed in an acetone solution containing 0.07 % β-mercaptoethanol. Proteins were extracted according to a method based on those described by Meyer and Pirovani [36, 37]. Samples were sonicated in an ultrasonic processor (GEX 130 130 W) to rupture basidiospores. Sonication was carried out as five 20-s pulses at 30-s intervals and 70 % amplitude. During the procedure, samples were kept on ice. After sonication, 800 μL of phenol, pH 8, was added and samples were vigorously vortexed for 2 min. Afterwards, they were centrifuged for 3 min at 10,000 × *g*. The upper phase (phenolic phase) was collected and transferred to a fresh tube, then five-fold (v/v) 100 % methanol with ammonium acetate 0.1 mol/L was added. Each sample was homogenized and stored at −20 °C for 30 min to precipitate protein. Proteins were recovered by centrifugation at 10,000 × *g* for 10 min. The precipitate was washed with cold 0.1 mol/L ammonium acetate in methanol and 80 % acetone twice, and then it was resuspended in a rehydration buffer (7 mol/L urea, 2 mol/L thiourea, 2 % CHAPS (3-3-Cholamidopropyl Dimethylammonio-1-Propanesulfonate), and 0.002 % Bromophenol blue). Proteins were quantified with a 2D-Quant kit (GE HealthCare), following manufacturer’s instructions (GE HealthCare) using bovine serum albumin (BSA) as a standard. Samples were stored at −20 °C until use.

### 1D and 2D-PAGE

For the 1D electrophoresis, 30 μg of protein from each treatment was separated in 12.5 % polyacrylamide gels using SDS-PAGE in an electrophoresis mini-tray (Omniphor) apparatus measuring 8 × 10 cm according to methods described by Laemmli [38].

For the 2D PAGE proteomic analysis, 350 μg of protein was used. Protein samples were applied to 13-cm gel strips (containing immobilines) with immobilized pH ranging from 3 to 10 non-linear (NL) (Amersham Biosciences, Immobiline™ Dry-Strip). IPG strips were then run in an Ettan IPGphor III Isoelectric Focusing Unit (GE HealthCare) with the following steps: 01:00 h at 500 Vh, 01:40 h at 1000 Vh, 2:30 h at 2,200 Vh, and 04:35 h at 800 Vh). The current was limited to 50 mA for each strip, and the temperature was kept at 20 °C for all focusing steps. After focusing, strips were incubated with 7 mL of equilibrium buffer containing 1 % DTT for 15 min with slow agitation. Afterwards, they were incubated with equilibrium buffer containing 2.5 % iodoacetamide with slow agitation for 15 min. Finally, the strips were washed with running buffer (0.025 mol/L Tris, pH 8.3, SDS 0.1 % and 0.19 mol/L glycine) for 15 min.

The second dimension was performed on a 12.5 % polyacrylamide gel in a HOEFER SE 600 Ruby vertical electrophoresis system (Amersham Bioscience). An initial electrical current of 15 mA/gel was applied for 15 min, after which 30 μA/gel was applied for 30 min, and then 50 μA/gel was applied for 3.5 h, totaling a 4.25 h run. Gels were run in triplicate for each germination time. After Coomassie Blue staining, proteins were unstained with distilled water for 5 days. Triplicates from each treatment were used for informatics evaluation using Image Master V.6 (GE Healthcare) software.

### Spot preparation and MS/MS analysis

All spots found in the ungerminated basidiospores sample gel were excised and processed according to methods described by Silva [22].

Peptides were resolved through reverse-phase chromatography in a nanoAcquity UPLC (WATERS) using two C_18_ columns, with the first being the “trapping” column (5 μm, 180 μm × 20 mm resin), and the second with a 1.7 μm, 100 μm × 100 mm resin, under a 0.6 μL/min flow for a 50-min run, and then 4 μL of each sample was collected. The peptides were separated according to their acetonitrile gradient; acetonitrile solutions were applied at these concentrations and times: 1 % for 1 min, from 1 to 50 % in 40 min, from 50 to 85 % in 5 min, 85 % for another 2 min, from 85 to 1 % in 1 min, and finally 1 % for 2 min, totaling a 50-min run. The separated peptides were ionized in a capillary unit under 3000 V (Micromass Q-TOFmicro), with fragments in the positive mode having a minimum relative intensity selection of 10 counts. The three most intense ions were analyzed by each 1-s scan, and collision energies ranged from 20 to 49 eV according to the mass/charge (*m/z*) ratio of the peptides.

### Identification of proteins

Spectra were processed and analyzed using ProteinLynx Global Server 4.2 software (WATERS) and compared to the NCBI database (http://www.uniprot.org/downloads, October 2012), using the MASCOT MS/MS Ion Search tool (www.matrixscience.com). Both databases were set for tryptic digestion, with one lost cleavage site, and cysteines modified through carbamidomethylation and methionine oxidation, tolerating error for 30 ppm peptides and mass error of MS/MS fragments of 0.1 Da. Only significant hits (*p* <0.05) were accepted according to the MASCOT analysis probability. After identification of the proteins, their ontology and biological function were verified with Uniprot (www.uniprot.org) and were organized into functional categories by BLAST2Go (www.blast2go.com).

### System biology analysis

In order to obtain information on the protein-protein interactions of ungerminated *M. perniciosa* basidiospores, orthologous sequences in *U. maydis* were used (Additional file 3: Table S4). *U. maydis* was chosen because it is a basidiomycota fungus that possesses the highest number of sequences orthologous to *M. perniciosa* due to the greater amount of annotated proteins and it is also a well-established model for studying dimorphism, virulence, plant-microbe interactions, and cell biology [39, 40]. In addition, the genome of this fungus has been annotated substantially [41]. Seventy-two true orthologous sequences (sequences which are found with the best hits) of *U. maydis* were obtained from the reciprocal BLASTp using amino acid sequences of *M. perniciosa* (http://blast.ncbi.nlm.nih.gov/Blast.cgi) with e-values approximately ≤ to 10^−10^ according to Moreno-Hagelsieb and Latimer [42]. The interaction network was analyzed through Cytoscape software version 2.8.3 [43]. The data were downloaded from the STRING 9.05 database (http://string-db.org). The following parameters were used for analysis: more than 50 interactions, significance level of 0.6, and addition of nodes until a saturated network was obtained. The MCODE plugin was used to cluster the proteins that had the highest relationships among themselves from the interaction force between them. The BiNGO plugin was used to analyze the biological processes, which were observed in the network in general.

## Results

### Basidiospore productivity

**Fig. 2:**
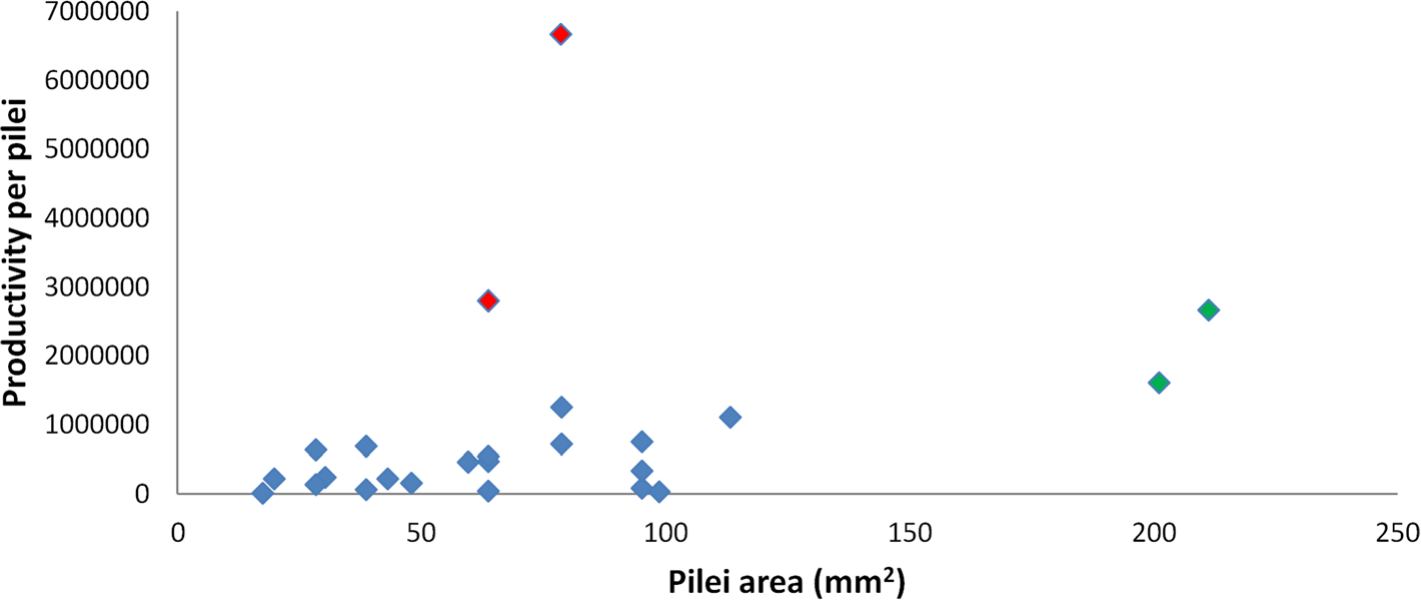
*Moniliophthora perniciosa* basidiospore productivity per pileus area. Red dots represent categories with more distant values compared to the average overall analysis (*black dots*)

In order to estimate the quantity of biological material for the production of basidiospores and the subsequent extraction of proteins, basidiocarps were collected and analyzed. We observed that the average pileus area ranged from 17.35 to 211.24 mm^2^. The minimum and maximum productivities were 23,200 and 6,666,667 basidiospores per basidiome, respectively (Fig. 2). The global spore productivity observed according to the average of amount of pilei per sample, was 744,477 basidiospores per basidiome. When four outlying samples were excluded from the data, an average productivity of 538,446 spores per basidiome was observed. The average productivity of basidiospores per pileus without the previously mentioned four outliers was 405,412 (Additional file 2: Table S1).

### Basidiospore germination and germ tube growth

**Fig. 3:**
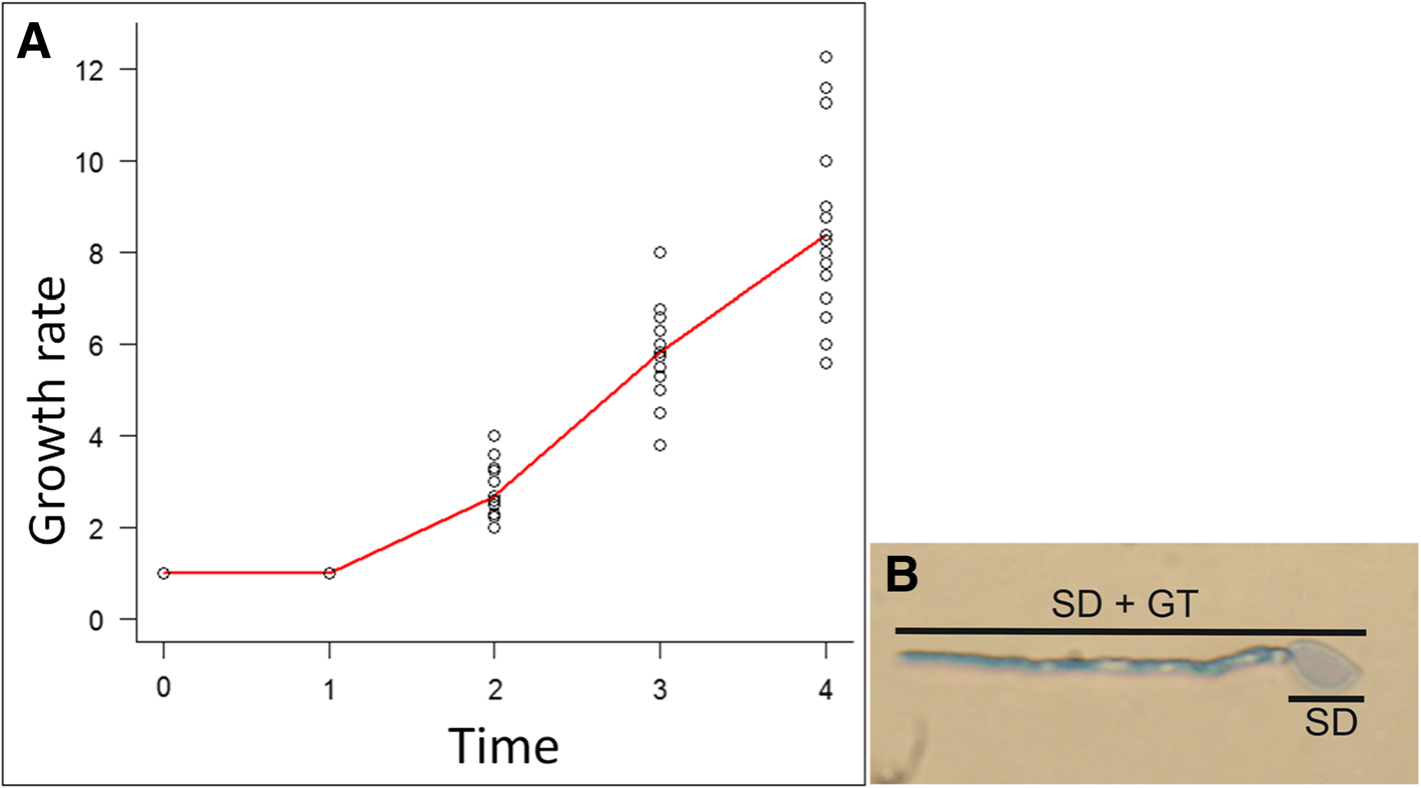
*Moniliophthora perniciosa* germ tube growth rate. **a** Growth rate of the spores during germination, expressed as the ratio between length of spore plus germ tube (SD+GT) and spore diameter (SD). [Ratio = (SD+GT)/SD]. **b** Growth scale of *M. perniciosa* basidiospores during germination. SD corresponds to the highest spore diameter, and SD+GT to spore length plus germ tube. The image was obtained 4 h after germination

At 4 h after germination was initiated, the ratio between germinated and ungerminated basidiospores was approximately 4:1 (Fig. 3a). After that period, spores were stained with Cotton Blue, and no dark-blue color was observed in the basidiospore bodies. However, an intense blue color was observed throughout the germ tubes (Fig. 3b).

### Protein yield

Based on the number of obtained spores and their relative protein mass, a protein yield estimate was conducted for each treatment (Table 1). The protein yields in micrograms per million basidiospores were approximately 0.161; 2.307, and 3.582 for germination times of 0, 2, and 4 HAG, respectively.

**Table 1:** Protein productivity of *M. perniciosa* ungerminated and germinated basidiospores

	Hours After Germination starts- HAG (treatments)		
	0 h	2 h	4 h
Number of basidiospores used	7 × 10^9^	5.2 × 10^8^	6.7 × 10^8^
Total protein obtained (μg)	1125	1200	2400
Protein yield for 10^6^ basidiospores (μg)	0.161	2.31	3.58
^a^Estimated number of pilei to produce 1050 μg of protein	16115	1122	723

Number of spores and protein yield

^a^Calculated considering the average productivity of 405.212 basidiospores per pileus for the one night-collection period (16 h of production). See Additional file 2: Table S1

### Protein extraction efficiency

**Fig. 4:**
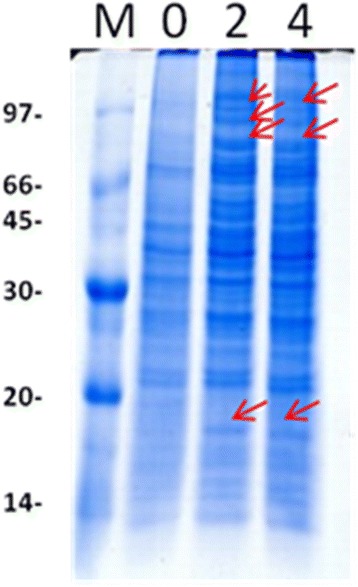
*Moniliophthora perniciosa* basidiospore protein profiles at different germination times. Arrows indicate bands with differential protein accumulation. M corresponds to the molecular mass marker, whose mass values in kDA are indicated on the left; 0, 2, and 4 correspond to the total protein extract purified at 0, 2, and 4 h after germination, respectively. Thirty micrograms of protein from each sample was resolved in 12.5 % SDS-PAGE and stained with colloidal Coomassie Blue (0.08 %)

In order to verify if the developed protein extraction method produced protein with high quality and integrity, we resolved 30 μg of each spore protein sample in a 12.5 % SDS-PAGE gel. Proteins were visualized with Coomassie Blue staining (Fig. 4). In all three treatments (0, 2, and 4 HAG), the observed protein profiles displayed well-resolved bands with molecular masses ranging from 10 to 200 kDa. Differences were observed between the 3 protein profiles; some bands were seen only after germination and other bands showed decreased intensity after germination (Fig. 4, arrows and dotted arrows, respectively). Throughout the protein profiles, variations in intensity of some bands can also be observed.

### 2D-PAGE analysis

**Fig. 5:**
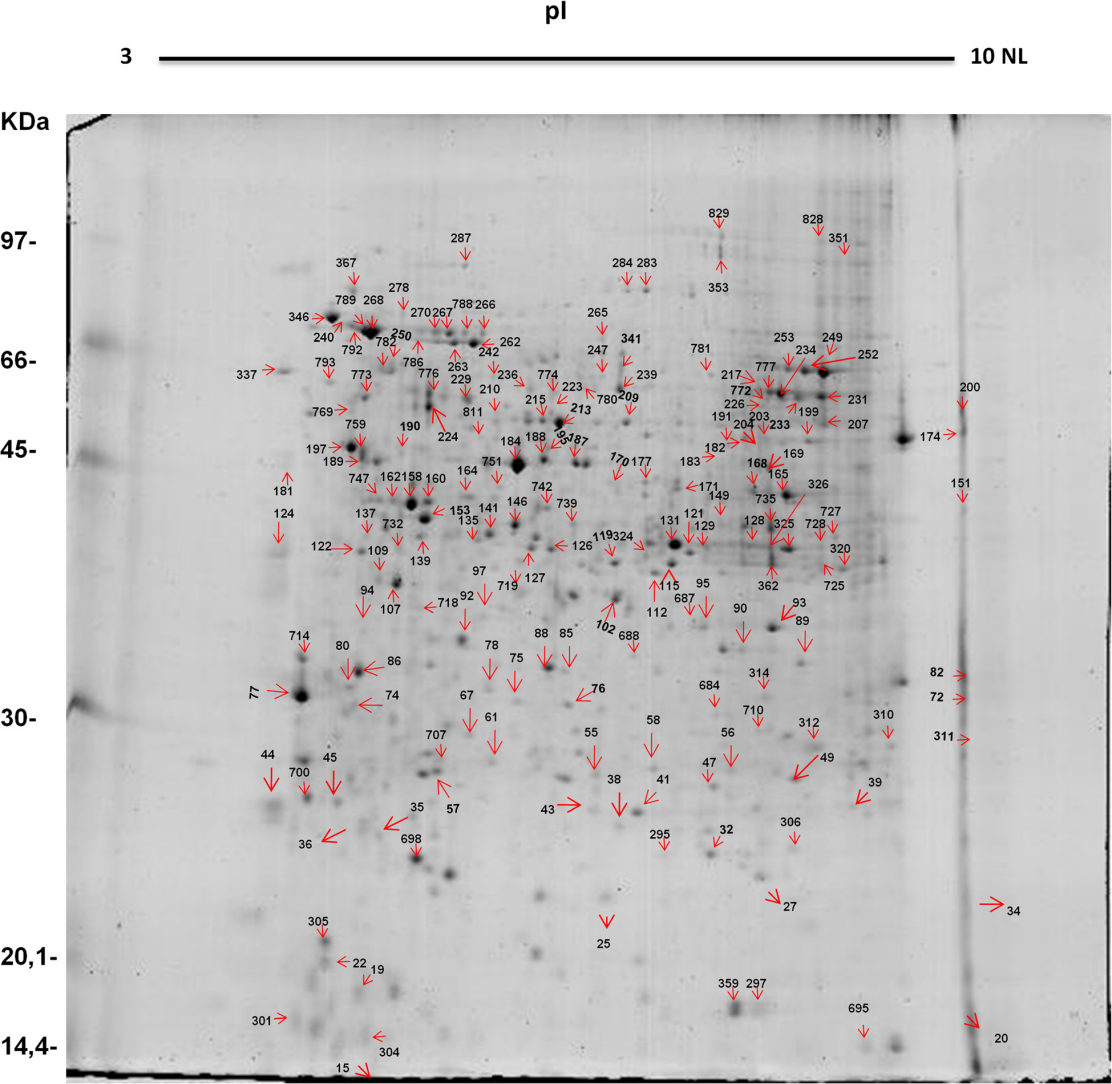
Protein profile in 2DE SDS-PAGE of ungerminated *M. perniciosa* basidiospores. The identity of spots is available in Additional file 5: Table S2. Replicate number 3 was used as a reference

We further analyzed the protein profile of *M. perniciosa* ungerminated spores (0 HAG) in a 2DE gel (Fig. 5). Samples were analyzed in triplicate. Protein spots were distributed in a broad non-linear pH range from 3 to 10. Gel analysis using Image Master V.6 (GE Healthcare) software revealed the presence of 549, 534, and 559 spots in gel replicates 1, 2, and 3, respectively. Most observed proteins (about 63.4 %) had a molecular weight between 30 and 60 kDa (data not shown). A dispersion graph was generated with a positive correlation of 0.95 between the SDS-PAGE replicates (Additional file 4: Figure S2).

### Identified proteins in ungerminated basidiospores

From the 2DE gel containing protein samples from ungerminated spores, a total of 400 spots were excised, processed, and analyzed using a mass spectrometer (Nano ESI Q-TOF, Micromass/Waters, Milford, MA, USA); 178 proteins were identified. Spots isolated from the 2DE gel are indicated with arrows in Fig. 5. The identification of the spots is shown in Additional file 5: Table S2.

**Fig. 6:**
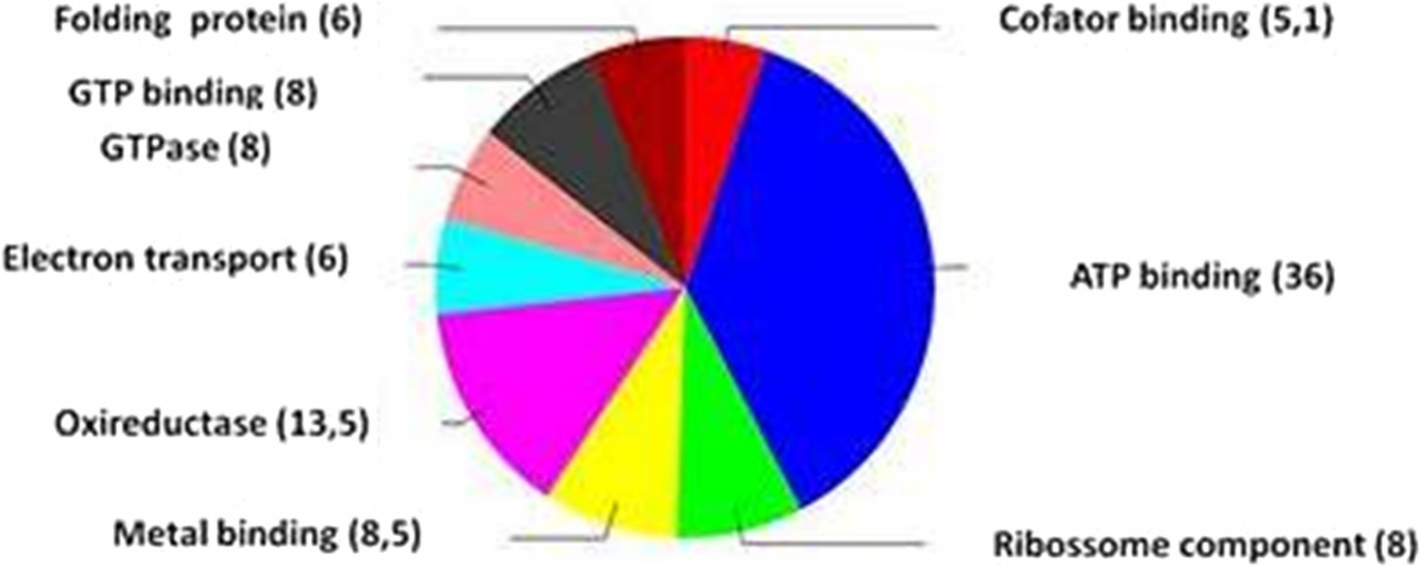
Representative functional classification of proteins that were expressed in *M. perniciosa* ungerminated basidiospores

The 178 proteins identified from the ungerminated basidiospores were classified into functional categories, which are displayed in Fig. 6. The category containing the highest number of identified proteins is related to energy processes (36 %), followed by proteins related to oxidoreductase activity (13.56 %), proteins linked to metal ions (8.57 %), ribosome structural components (8 %), proteins related to GTP linkage (8 %), electron-carrying proteins (6 %), proteins with GTPase activity (6 %), proteins related to protein folding (6 %), and co-factors (5.16 %).

### Description of the interaction network

**Fig. 7:**
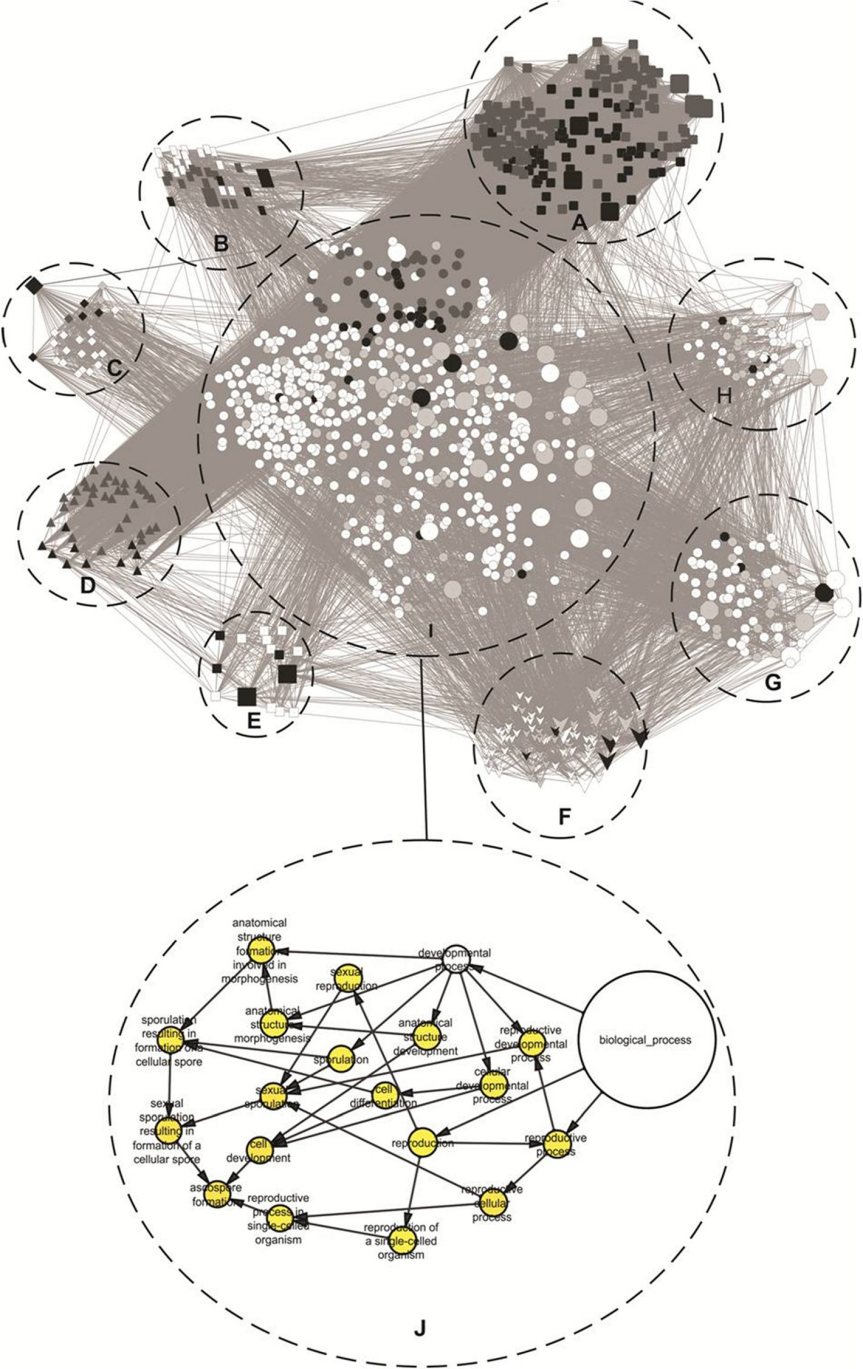
Interaction network of proteins in *Moniliophthora perniciosa* basidiospores. **a** Cluster 1: proteins that are related to protein synthesis and degradation. **b** Cluster 2: proteins that are related to cell metabolism. **c** Cluster 3: proteins that are related to cell respiration. **d** Cluster 4: proteins that are involved in RNA processing. **e** Cluster 5: proteins that are related to gene transcription and expression. **f** Cluster 6: proteins that are involved in ion transportation. **g** Cluster 7: proteins that are involved in cytoskeleton regulation and organization. **h** Cluster 8: proteins that are involved in amino acid biosynthesis. **i** Unclustered proteins. Each geometric shape represents a group, as separated by its MCODE. Bigger sized shapes represent orthologous proteins that were identified in this study. Dark, medium, and light gray tones represent the proteins that are considered hub-bottlenecks, hub, and bottlenecks, respectively. **j** Representations of the main biological processes that are performed by proteins that were not grouped by BiNGOA plugin. The arrows represent the ranking of biological processes from most general to most specific

The protein-protein interaction network that used orthologous proteins of *U. maydis* displayed the presence of 1066 proteins (nodes) and 22,769 connectors, with a confidence level of 0.6. Of the 72 orthologous sequences of proteins found from the reciprocal BLAST with *U. maydis*, 66 were located within the network (Additional file 3: Table S4) and they are represented in higher node sizes (Fig. 7). A survey of the gene ontology for the biological processes of all proteins in the network was performed using the BiNGO plugin.

A cluster analysis was performed with the MCode plugin, which resulted in clusters with scores higher than 2.5 (Fig. 7a-h; Additional file 6: Table S3). The main cluster (cluster 1) represented proteins related to protein synthesis or degradation. This cluster contains 178 proteins and 8,157 connectors. Seven orthologous proteins identified in this cluster (RPS1, UM00868.1, UM05990.1, RPS0, UM03578.1, UM01318.1 and UM04986.1) also interact with other proteins. Those interacting proteins are highlighted with bigger circles inside the cluster (Fig. 7a). Cluster 2 represents 35 proteins and 519 connectors. Two orthologous proteins are part of that cluster (UM02562.1 and tef1). Protein UM02562.1 is a hub (represented by the medium gray color), and it interacts with several other proteins that are associated with cell metabolism (Fig. 7b). Cluster 3 contains 25 proteins and 298 connectors. In this cluster, only one orthologous protein was grouped—UM05993.1—a mitochondrial protein represented as a hub-bottleneck which interacts with many other proteins associated with cellular respiration (Fig. 7c). Cluster 4 presents 43 proteins and 416 connectors. All proteins in this cluster are related to biosynthesis and RNA processing. No orthologous proteins identified in this study were grouped in this cluster (Fig. 7d). Cluster 5, the smallest cluster, contained only 15 proteins, two of which were orthologous proteins identified through MS/MS. Orthologous protein UM00157.1 is a pyruvate kinase, and UM02776.1 is a nucleoside diphosphate kinase that activates transcription. Those proteins were considered to be hub-bottlenecks, and they interact with many other proteins involved in transcriptional processing. Eighty proteins and 422 connectors form cluster 6, which cluster with the highest number of orthologous proteins: 10 (UM05090.1, UM04871.1, UM03951.1, UM00621.1, UM03356.1, UM03527.1, UM04971.1, UM04562.1, UM00595.1, UM02462.1). Four of those proteins were considered to be hub-bottlenecks, and they interact with xmany other proteins that are involved in electron transport (Fig. 7f). Cluster 7 represented 75 proteins and 242 connectors. Seven orthologous proteins from the initial study were grouped in this cluster (UM01672.1, UM0068.1, UM00403.1, UM05379.1, UM05918.1, UM06453.1, UM04507.1). Protein UM00403.1 is considered a hub-bottleneck and seems to be a key protein for cell regulation, mainly for the regulation of actin filament polymerization (Fig. 7g). Cluster 8 has 47 proteins and 117 connectors. Four original orthologous proteins were placed in this group (UM02715.1, UM028991.1, UM03449.1, UM03734.1). Most proteins in this interaction group are involved in the biosynthesis of amino acids and cell division (Fig. 7h; Additional file 6: Table S3). Thirty-three orthologous proteins identified in this study were not found in any of the clusters (Fig. 7i). However, the analysis shows an interaction of proteins involved in important processes for the fungus, such as reproduction and primary hypha development (Fig. 7j). Proteins such as UM01366.1, UM02442.1, UMS2, UM06217.1 directly interact with each other and with several other proteins involved in chitin wall formation and filament development. Three unclustered proteins were considered as hub-bottlenecks. They are UM06217.1, tub1, and UM02442.1. UM06217.1 is an actin protein, which directly interacts with nine other proteins in this study and 130 other proteins distributed all over the network (Additional file 7: Figure S3).

## Discussion

This is the first study of the characterization of *M. perniciosa* basidiospore proteins, probably due to the difficulty in obtaining protein from spores in sufficient quantity and homogeneity to conduct proteomic studies. During the production of basidiocarps for this study, the productivity of basidiospores per pileus was analyzed for different basidiomes size categories (which were expressed per area in Fig. 2).

We have shown that the yield of basidiospores per pileus is highly variable, but the average is approximately 400,000 basidiospores per pileus during a one-night collection. Considering the protein yield for each germination time-point, the number of basidiomes needed to conduct proteomic analyses was estimated. The results showed increasing protein yield of *M. perniciosa* basidiospores during the germination process (Table 1). The final protein mass obtained for ungerminated spores was enough to prepare three replicates of the 2DE gel, as each replicate requires 350 μg. A much larger number of basidiomes is required to obtain enough protein to analyze ungerminated basidiospores in comparison to germinated ones (approximately 222 times more), according to the estimated protein yield that was assessed for germination times of 2 and 4 h. Some fungal spores require protein synthesis during the early development phase so biosynthesis of essential enzymes involved in respiratory pathways can occur [44–46]. The high number of spores released per pileus may be related to the high dispersion power of the fungus, but the determining factor for the epidemiology of the disease is the presence or absence of conditions such as host phenology and basidiocarp production [47]. Furthermore, the interaction between plant characteristics and a favorable climate (alternating dry and humid periods) is of fundamental epidemiological importance for this disease [48].

Around 90 % of the basidiospores stored in 16 % glycerol germinated when the solution was diluted to a glycerol concentration of 3 %, similar to observations by Frias et al., [8]. This can be explained by the fact that a glycerol concentration of 16 % keeps H_2_O from entering the basidiospore, hindering germination. The swelling after glycerol concentration was reduced to 3 % may be explained by the inflow of water from the external environment [49]. Trehalose degradation and glycerol biosynthesis in other fungi (such as yeasts and *A. nidulans*) increase the internal osmotic potential, which favors water absorption [50, 51]. In that context, we have identified three orthologous proteins that were not clustered in the interaction network. These proteins are GTPases UM05511.1 and UM00756.1, and actin UM06217.1, which directly interacts with MAP Kinase HOG1 (high-osmolarity glycerol). The HOG pathway was first discovered in the yeast *Saccharomyces cerevisiae*, and it seems to be activated to regulate hyperosmotic stress [52]. Tolerance to hyperosmotic stress may be a fundamental process in the life cycle of filamentous fungi [53].

As most fungi have a resistant and rigid cell wall, the protein extraction process is a key step for proteomic studies of these microorganisms [24]. Basidiospore protein extraction with phenol seemed very efficient, and resulted in well-resolved and well-defined bands with no drag, indicating a lack of interfering compounds as seen in the SDS-PAGE gel (Fig. 4). Extraction with phenol resulted in high quantities of soluble protein and good reproducibility in both the first and second dimensions (Fig. 5). An efficient phenol-based extraction solubilizes membrane proteins, removes nucleic acids, and minimizes proteolysis.

Taking into account the difficulty in obtaining the biological samples, we may consider the quantity (178) of proteins identified through mass spectrometry to be satisfactory, and within the averages found by other authors. Noir et al., identified 180 proteins by 2DE analysis of *Blumeria graminis* conidiospore spots, the phytopathogenic fungus responsible for barley mildew [25]. Quin et al., identified 24 conidium proteins in entomopathogenic fungus *Nomuraea rileyi* isolated from infected silkworms [54] and Barros et al., identified 65 proteins in conidia of the entomopathogenic fungus *Metarhizium acridum* to build a reference map [55].

Identical or similar functional classifications for different spots are common in proteomic studies that use 2DE SDS-PAGE [25]. This may be caused by post translational modifications (PTMs) in products of the same gene, or the presence of protein isoforms coded for different genes [56]. Phosphorylation is a very common and dynamic PTM in cell biochemical processes. Phosphorylation can regulate increases or decreases in enzyme activity, support protein migration to other cell compartments to allow for protein-protein interaction, and also mark proteins for degradation [57].

### Identified proteins and interaction network

Proteomic analysis using systems biology may give us a broad view of the main process, which occur in fungi before they invade plant tissues. Most proteins we identified in the basidiospore are involved in cell energy metabolism (Fig. 6). This may also be visualized by the 10 orthologous proteins that are part of cluster 6, all of which are related to cell metabolism (Fig. 7h).

Metabolic polypeptides are much more abundant in soluble protein samples, and are well represented in proteomic studies. This also applies to fungal proteome analyses, for example, where proteins involved in metabolic processes accounted for approximately 66 % of the total proteins identified in uredospores of phytopathogenic basidiomycete *Uromyces appendiculatus* [58]. Similarly, proteins responsible for metabolism during the germinative process accounted for 75 % of the total proteins identified in the analysis of conidiospores of *Blumeria graminis* [25].

Within the protein group that relates to cell metabolism, cluster 7, four proteins were considered to be hub-bottlenecks (UM04971.1, fumarate reductase; UM04562.1, aspartate aminotransferase; UM02461.1, dihydrolipoyl dehydrogenase; and UM00595.1, aspartate aminotransferase). Those proteins play a key role in the citric acid cycle.

Although the majority of identified proteins are not related to protein synthesis or degradation (Fig. 6), these proteins represent the largest cluster in the interaction network (Fig. 7a). Seven orthologous ribosome proteins were found. These proteins were grouped in this cluster (RPS1, UM00868.1, UM05990.1, RPS0, UM03578.1, UM01318.1, UM04986.1), and they also interact with other proteins. Ribosome protein UM04986.1 is the hub-bottleneck that makes the most direct interactions. It is involved in 182 direct interactions with proteins that are mainly distributed with proteins from cluster 4, and some unclustered ones (Additional file 8: Figure S4). Among those are proteins involved in RNA transcription and processing (PB1, UM06156.1, UM06331.1, UM05334.1, UM03058.1, UM02324.1, UM02903.1, UM04722.1).

Proteins considered as hub-bottlenecks, associated in cluster 5, play a key role in transcriptional processes (RPB1; UM03988.1). This may be related to the demand for proteins that are responsible for filament development. For example, protein UM00157.1, a hub-bottleneck protein in group 5, is a pyruvate kinase that directly interacts with key proteins related to hypha growth and development of filamentous fungi such as protein UAC1, an adenylate cyclase that was isolated and classified in *U. maydis* (Additional file 9: Figure S5). Haploid lineages of *U. maydis*, in which the UAC1 gene was deleted, exhibit a stable filamentous phenotype and loss of pathogenic capacity [59].

Half of the orthologous proteins (33) identified in this study were not clustered in the network, but several of them play a very important role in hypha development and branching. Actin UM06217.1 is considered a hub-bottleneck protein, and it directly interacts with 139 other proteins that are distributed all over the network (Additional file 7: Figure S3). Among the proteins that are part of that interaction, some of them deserve special attention for being very important in the invasive growth of hyphae. One such protein is CDC42. Yeast mutants ectopically expressing CDC42, were unable to form invader hyphae filaments in response to high temperature [60]. Another protein deserving attention is UAC1. When the gene coding for this protein was deleted in *U. maydis*, the mutant strains displayed a branched hyphal phenotype and a loss of fungal pathogenicity [61]. RAC1 expression is involved with the shape of the septum and hypha growth in *U. maydis* [62]. The actin UM06217.1 also is able to interact with the MAP kinase CHK1, which operates in DNA damage repair checkpoints during G2, and plays a key role in the regulation of the cell cycle [63]. Finally, protein KPP6 should be mentioned. According to Brachmann [64], mutant strains deleted for this gene may form normal appressoria, but are unable to penetrate into the plant cuticle. KPP6 may respond to a plant signal by regulating the necessary genes for efficient penetration into the plant tissue. These aforementioned proteins are part of fundamental processes such as sporulation, reproduction, and cell differentiation for the successful start of spore germination on the surface of plants (Fig. 7i). The interactions among these proteins may provide insights on how the molecules play during the early fungal development and indicates target proteins to drug development and protein activity inhibition in order to decrease the *M. perniciosa* pathogenicity and disease control.

## Conclusions

In summary, in this study, we showed that the productivity of *M. perniciosa* basidiospores per pileus area is highly variable, but the average is 405.212 basidiospores per pileus during a one-night collection. The protein yield per spore increases during the 4 h after germination. The numbers of basidiospores 7 × 10^9^, 5.2 × 10^8^, and 6.7 × 10^8^ obtained in the 0, 2, and 4 HAG groups, respectively, are sufficient to obtain enough protein mass for the three 2D-PAGE replicates. The protein extraction method that is based on sedimentation, sonication with dense SDS, and phenolic extraction, which was utilized in this study, was effective, presenting good resolution and reproducibility for *M. perniciosa* basidiospores.

We also present the first 2D-PAGE reference map of proteins expressed during germination, showing the protein types that are likely involved in fungal development. This fungal developmental stage is very important, as it represents the first contact between plant and pathogen. We identified orthologous proteins in *U. maydis* such as hub-bottleneck ribosome protein UM04986.1, actin UM06217.1, and pyruvate kinase UM00157.1, the interaction of which helps play a very important role in hypha development and branching. The identification of proteins contained in the reference gel elucidated many potential molecular interactions involved in the initial metabolic processes during fungal development.

In addition, this protein network may also be used to determine proteins involved in fungal and phytopathogenic processes that are candidates for targeting with disease-controlling drugs. This information guides the planning of experiments that involve proteome analyses of *M. perniciosa* fungus basidiospores.

## Abbreviations

BOD, biochemical oxygen demand; CEPEC, Centro de Pesquisas do Cacau; CEPLAC, Comissão Executiva do Plano da Lavoura Cacaueira; HAG, hours after germination; SDS-PAGE, sodium dodecyl sulfate-Polyacrylamide gel electrophoresis

## Additional files

Additional file 1: Figure S1. Workflow to present the steps taken in this study. (JPG 1055 kb) Additional file 2: Table S1. Basidiospore production and protein yield analyzed. (XLS 48 kb) Additional file 3: Table S4 Orthologous sequences of genes from *Ustilago maydis* used to construct the network along with their annotations. (XLS 33 kb) Additional file 4: Figure S2. Dispersion graph among the SDS-PAGE replicates. (TIF 489 kb) Additional file 5: Table S2. Identification of spots marked with arrows on the reference gel. (XLS 213 kb) Additional file 6: Table S3. Gene ontology for the biological processes of all proteins in the network using BiNGOA plugin, and the cluster analysis performed with the MCode plugin. (XLSX 145 kb) Additional file 7: Figure S3. Interaction of actin UM06217.1 which directly interacts with nine other proteins in this study and other 130 proteins distributed all over the network. (TIF 1163 kb) Additional file 8: Figure S4. Direct interactions of the ribosome hub-bottleneck protein UM04986.1. (TIF 1240 kb) Additional file 9: Figure S5. Interaction of hub-bottleneck protein UM00157.1 with key proteins related to hypha growth and development of filamentous fungi protein UAC1. (TIF 1045 kb)
