# Supplementary figures and images for: Protein profile and protein interaction network of Moniliophthora perniciosa basidiospores
Source: BMC Microbiol. 2016 Jun 24;16:120. doi: 10.1186/s12866-016-0753-0 (PMC4919874; doi:10.1186/s12866-016-0753-0)

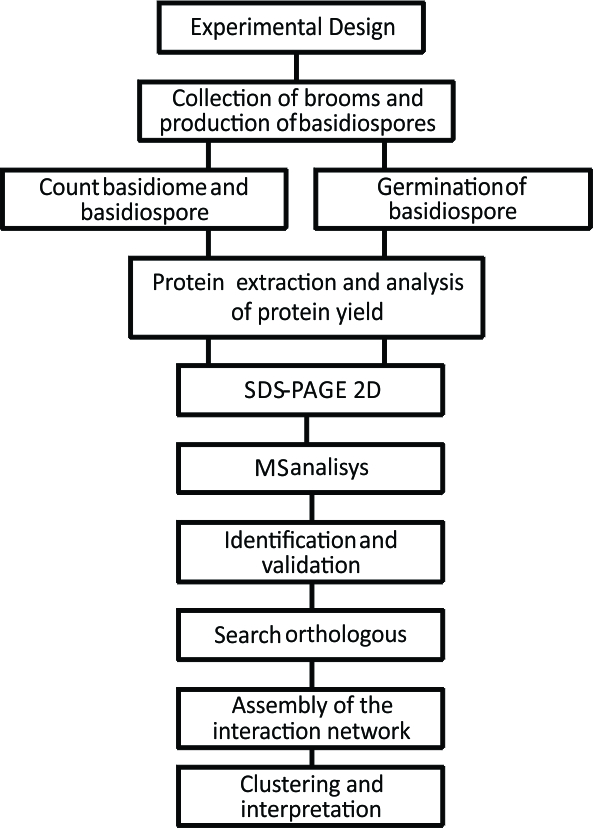

Supplement: Additional file 1: Figure S1. — Workflow to present the steps taken in this study. (JPG 1055 kb) [file 12866_2016_753_MOESM1_ESM.jpg]

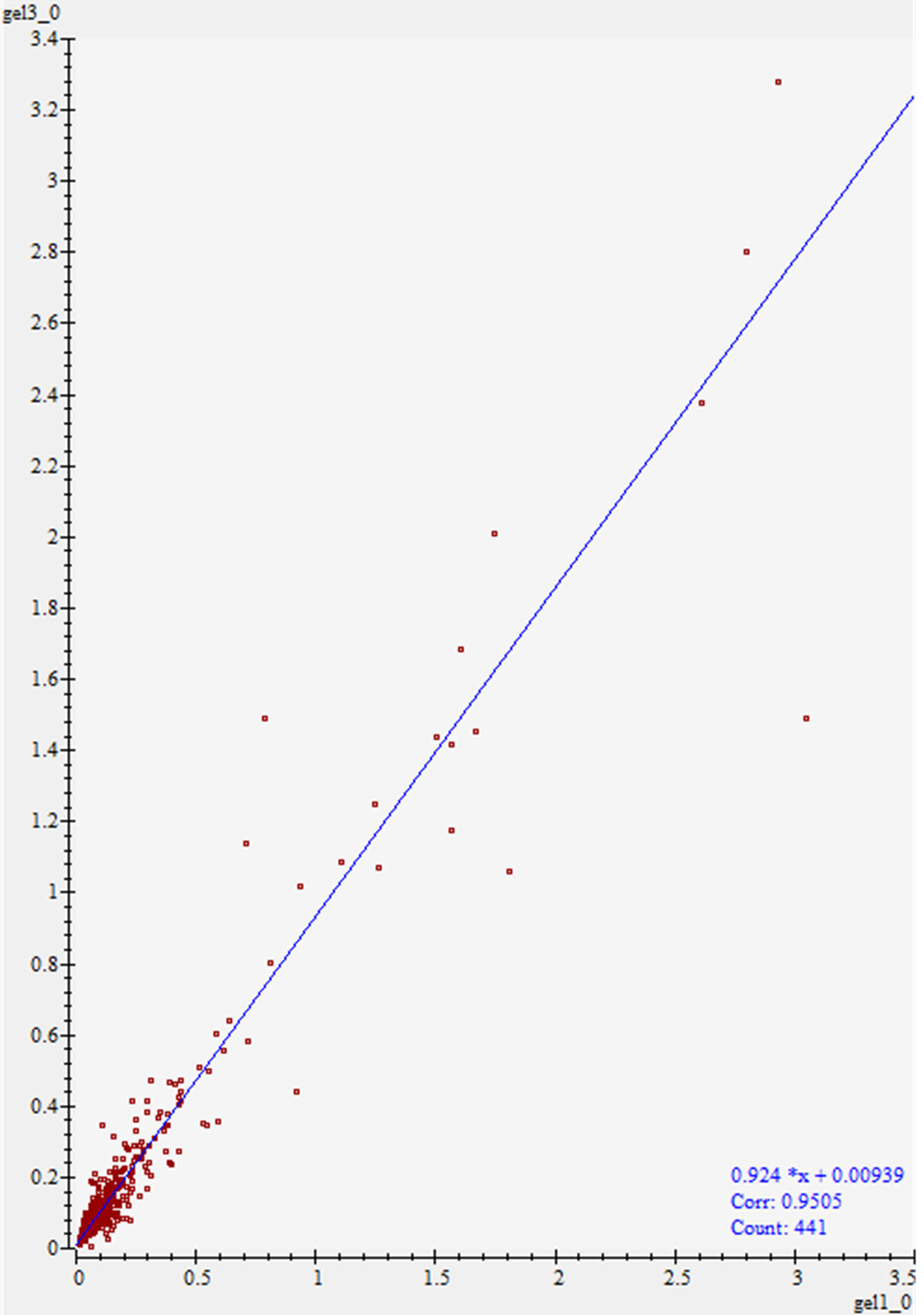

Supplement: Additional file 4: Figure S2. — Dispersion graph among the SDS-PAGE replicates. (TIF 489 kb) [file 12866_2016_753_MOESM4_ESM.tif]

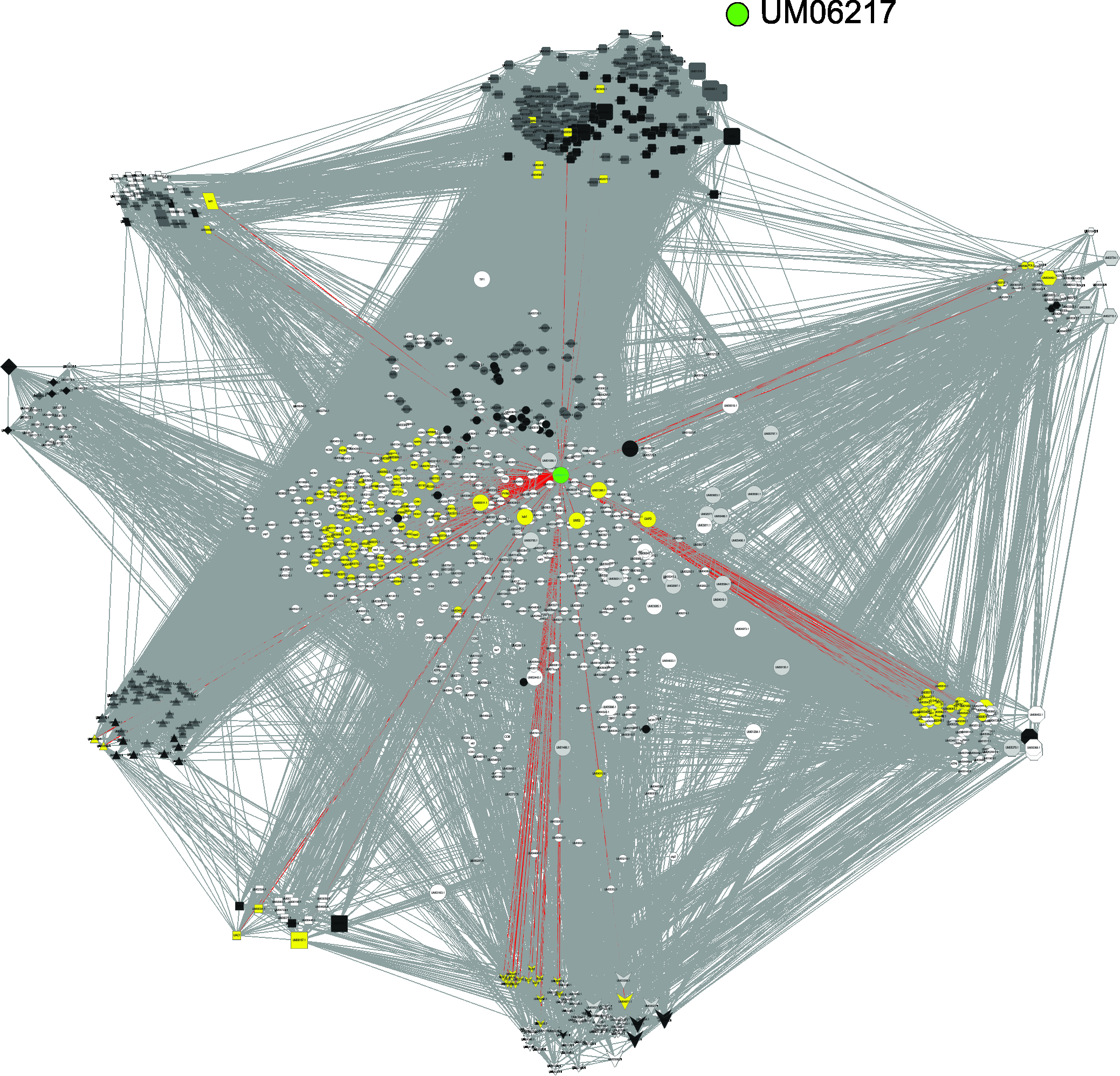

Supplement: Additional file 7: Figure S3. — Interaction of actin UM06217.1 which directly interacts with nine other proteins in this study and other 130 proteins distributed all over the network. (TIF 1163 kb) [file 12866_2016_753_MOESM7_ESM.tif]

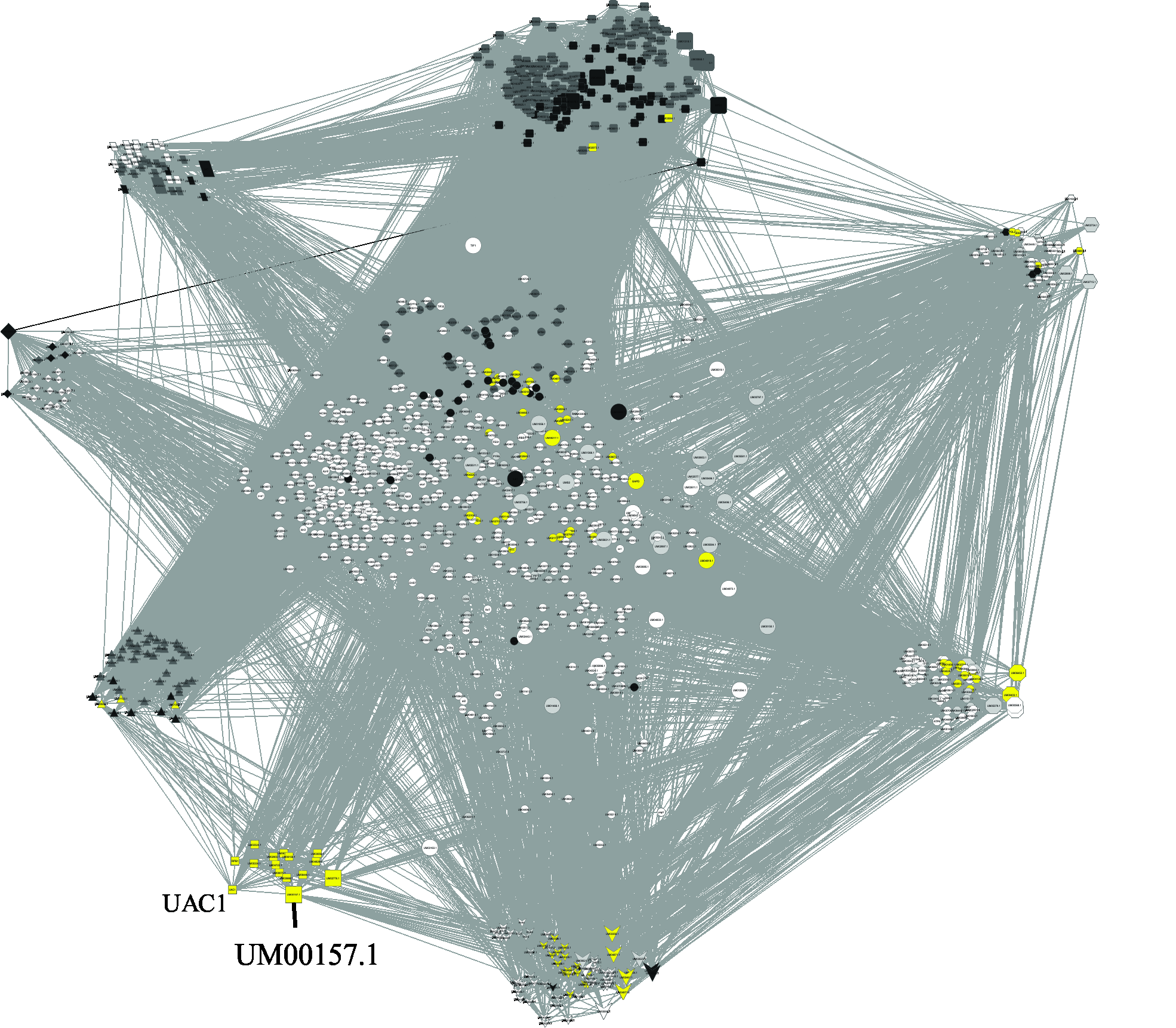

Supplement: Additional file 8: Figure S4. — Direct interactions of the ribosome hub-bottleneck protein UM04986.1. (TIF 1240 kb) [file 12866_2016_753_MOESM8_ESM.tif]

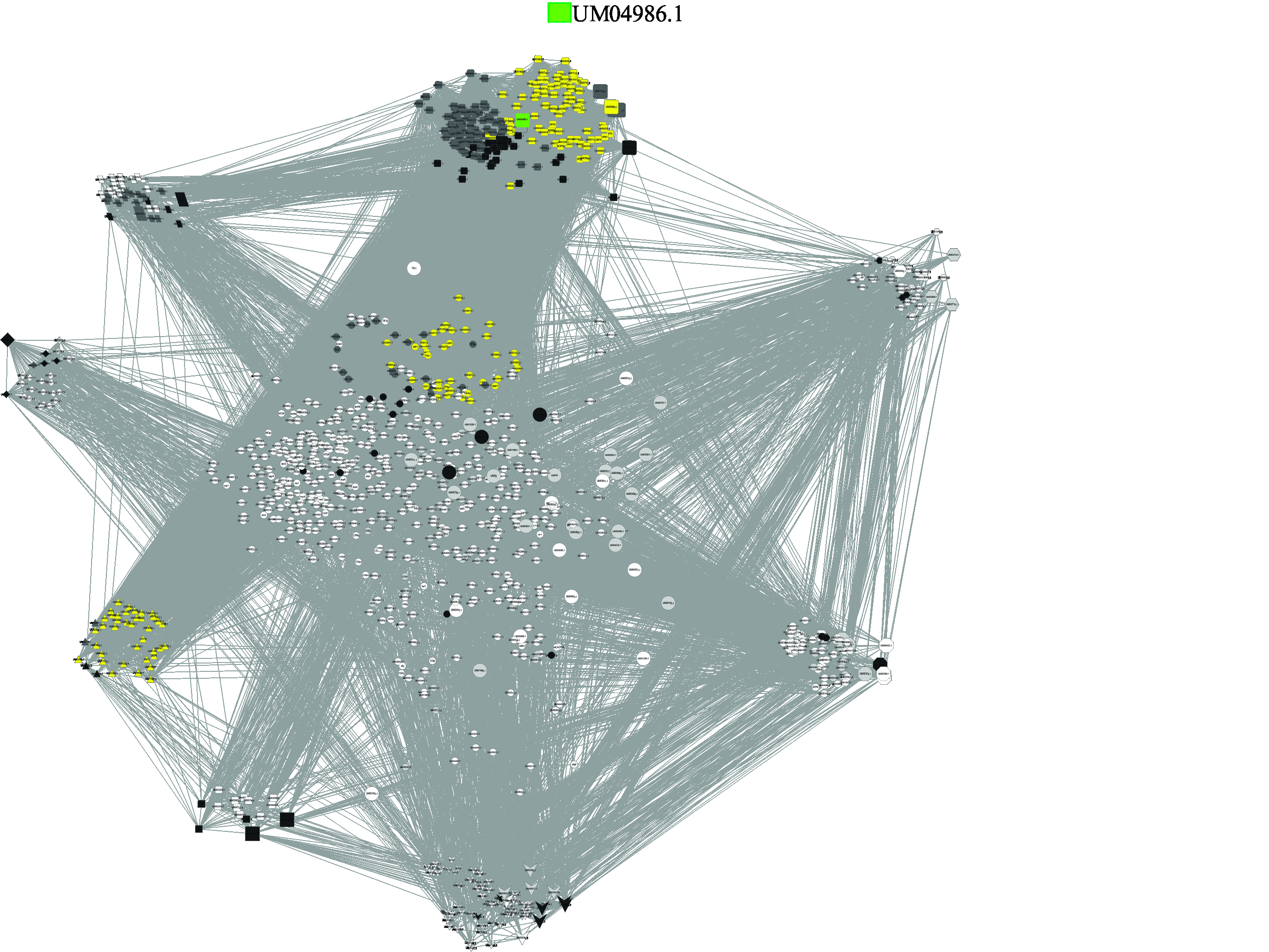

Supplement: Additional file 9: Figure S5. — Interaction of hub-bottleneck protein UM00157.1 with key proteins related to hypha growth and development of filamentous fungi protein UAC1. (TIF 1045 kb) [file 12866_2016_753_MOESM9_ESM.tif]
